# Supplementary material for: A direct comparison of theory-driven and machine learning prediction of suicide: A meta-analysis
Source: PLoS One. 2021 Apr 12;16(4):e0249833. doi: 10.1371/journal.pone.0249833 (PMC8041204; doi:10.1371/journal.pone.0249833)
Supplement: S1 Table — (DOCX) [file pone.0249833.s002.docx]

Supporting Information

S1 Table. Model Related Search Terms

| emotion regulation | biology |
| --- | --- |
| emotional regulation | hormones |
| emotion dysregulation | neurological |
| emotion control | molecules |
| psychopathology | cells |
| internalizing | genetics |
| externalizing | hopelessness |
| ideation to action | hope |
| thwarted belongingness | apathy |
| perceived burdensomeness | pessimism |
| acquired capability | complex |
| increased pain tolerance | machine learning |
| lowered fear of death | artificial intelligence |
| interpersonal theory | data mining |
| motivational volitional | computer applications |
| 3 step theory | data processing |
| connectedness | computer software |
| social support | Automated Information Processing |
| alienation | expert systems |
| isolation | pattern recognition |
| belonging | machine systems |
| burden | case based reasoning |
|  | human machine systems |

Presented in this Supplemental Section are the search terms used for the present meta-analysis. Search terms listed in the “Model Related Search Terms” are related to theoretically-driven or machine learning models. Each search term from the “Model Related Search Terms” list was paired with a search term from the “STB Outcome Search Terms” as well as the “Longitudinally Relevant Search Terms” to achieve strings of search terms.
