# Supplementary material for: A direct comparison of theory-driven and machine learning prediction of suicide: A meta-analysis
Source: PLoS One. 2021 Apr 12;16(4):e0249833. doi: 10.1371/journal.pone.0249833 (PMC8041204; doi:10.1371/journal.pone.0249833)
Supplement: S2 Table — (DOCX) [file pone.0249833.s003.docx]

S2 Table. Suicide-Related Outcome Search Terms

| suicide | suicidality |
| --- | --- |
| suicide attempt | suicide death |
| suicide ideation | suicide risk |
| suicidal |  |

Presented in this Supplemental Section are the search terms used for the present meta-analysis. Search terms listed in the “Model Related Search Terms” are related to theoretically-driven or machine learning models. Each search term from the “Model Related Search Terms” list was paired with a search term from the “STB Outcome Search Terms” as well as the “Longitudinally Relevant Search Terms” to achieve strings of search terms.
