# Supplementary material for: A direct comparison of theory-driven and machine learning prediction of suicide: A meta-analysis
Source: PLoS One. 2021 Apr 12;16(4):e0249833. doi: 10.1371/journal.pone.0249833 (PMC8041204; doi:10.1371/journal.pone.0249833)
Supplement: S3 Table — (DOCX) [file pone.0249833.s004.docx]

S3 Table. Longitudinally Relevant Search Terms

| longitudinal | prospective |
| --- | --- |
| predictive | prediction |
| predicts | long term |
| over time | future |

Presented in this Supplemental Section are the search terms used for the present meta-analysis. Search terms listed in the “Model Related Search Terms” are related to theoretically-driven or machine learning models. Each search term from the “Model Related Search Terms” list was paired with a search term from the “STB Outcome Search Terms” as well as the “Longitudinally Relevant Search Terms” to achieve strings of search terms.
