# Supplementary material for: A direct comparison of theory-driven and machine learning prediction of suicide: A meta-analysis
Source: PLoS One. 2021 Apr 12;16(4):e0249833. doi: 10.1371/journal.pone.0249833 (PMC8041204; doi:10.1371/journal.pone.0249833)
Supplement: S1 File — (DOCX) [file pone.0249833.s005.docx]

Supplemental References

1. *Abbar, M., Courtet, P., Bellivier, F., Leboyer, M., Boulenger, J. P., Castelhau, D., ... & Vessaz, M. (2001). Suicide attempts and the tryptophan hydroxylase gene. *Molecular psychiatry*, *6*(3), 268. http://dx.doi.org/10.1038/sj.mp.4000846

2. *Asberg, M., Träskman, L., & Thorén, P. (1976). 5-HIAA in the cerebrospinal fluid. A biochemical suicide predictor? Archives of General Psychiatry, 33, 1193–1197.

3. *Batterham, P. J., Walker, J., Leach, L. S., Ma, J., Calear, A. L., & Christensen, H. (2018). A longitudinal test of the predictions of the interpersonal-psychological theory of suicidal behaviour for passive and active suicidal ideation in a large community-based cohort. *Journal of Affective Disorders*, *227*, 97–102. https://doi.org/10.1016/j.jad.2017.10.005

4. *Beautrais, A. L. (2003). Subsequent mortality in medically serious suicide attempts: a 5 year follow-up. *Australian & New Zealand Journal of Psychiatry*, *37*(5), 595–599. https://doi.org/10.1046/j.1440-1614.2003.01236.x

5. * Bennardi, M., Caballero, F. F., Miret, M., Ayuso, M. J. L., Haro, J. M., Lara, E., … Ayuso-Mateos, J. L. (2019). Longitudinal Relationships Between Positive Affect, Loneliness, and Suicide Ideation: Age-Specific Factors in a General Population. *Suicide & Life-Threatening Behavior*, *49*(1), 90–103. https://doi.org/10.1111/sltb.12424

6. *Beck, A. T., Steer, R. A., & Trexler, L. D. (1989). Alcohol abuse and eventual suicide: A 5- to 10-year prospective study of alcohol-abusing suicide attempters. *Journal of Studies on Alcohol*, 50, 202–209

7. *Beck, A. T., Steer, R. A., Kovacs, M., & Garrison, B. (1985). Hopelessness and eventual suicide: A 10-year prospective study of patients hospitalized with suicidal ideation. *The American Journal of* Psychiatry, 142, 559–563.

8. *Berglund, M. (1984). Suicide in alcoholism. A prospective study of 88 suicides: I. The multidimensional diagnosis at first admission. Archives of General Psychiatry, 41,888–891

9. * Black, D., Monahan, P., & Winokur, G. (2002). The Relationship Between DST Results and Suicidal Behavior. *Annals of Clinical Psychiatry (Springer Science & Business Media B.V.)*, *14*(2), 83–88. https://doi.org/10.3109/10401230209149094

10. *Blumenthal, S., Bell, V., Neumann, N. U., Schüttler, R., & Vogel, R. (1989). Mortality and rate of suicide of first admission psychiatric patients. A 5-year follow-up of a prospective longitudinal study. *Psychopathology*, 22, 50–56.

11. *Brent, D. A., Melhem, N. M., Oquendo, M., Burke, A., Birmaher, B., Stanley, B., ... & Porta, G. (2015). Familial pathways to early-onset suicide attempt: a 5.6-year prospective study. *JAMA psychiatry*, *72*(2), 160-168. https://doi.org/10.1001/jamapsychiatry.2014.2141

12. * Burke, T., Connolly, S., Hamilton, J., Stange, J., Abramson, L., Alloy, L., … Alloy, L. B. (2016). Cognitive Risk and Protective Factors for Suicidal Ideation: A Two Year Longitudinal Study in Adolescence. *Journal of Abnormal Child Psychology*, *44*(6), 1145–1160. https://doi.org/10.1007/s10802-015-0104-x

13. *Cheng, Q., Li, T. M., Kwok, C. L., Zhu, T., & Yip, P. S. (2017). Assessing suicide risk and emotional distress in Chinese social media: A text mining and machine learning study. *Journal of medical internet research*, *19*(7), e243.

14. * Chiu, Y.-C., Tseng, C.-Y., & Lin, F.-G. (2017). Gender differences and stage-specific influence of parent–adolescent conflicts on adolescent suicidal ideation. *Psychiatry Research*, *255*, 424–431. https://doi.org/10.1016/j.psychres.2017.06.077

15. *Choi, S. B., Lee, W., Yoon, J. H., Won, J. U., & Kim, D. W. (2018). Ten-year prediction of suicide death using Cox regression and machine learning in a nationwide retrospective cohort study in South Korea. *Journal of affective disorders*, *231*, 8-14.

16. * Chu, C., Hom, M. A., Rogers, M. L., Stanley, I. H., Ringer-Moberg, F. B., Podlogar, M. C., … Joiner, T. E. (2017). Insomnia and suicide-related behaviors: A multi-study investigation of thwarted belongingness as a distinct explanatory factor. *Journal of Affective Disorders*, *208*, 153–162. https://doi.org/10.1016/j.jad.2016.08.065

17. * Consoloni, J. L., Lefebvre, M. N., Zendjidjian, X., Olié, E., Mazzola-Pomietto, P., Desmidt, T., ... & Haffen, E. (2018). Serotonin transporter gene expression predicts the worsening of suicidal ideation and suicide attempts along a long-term follow-up of a Major Depressive Episode. *European Neuropsychopharmacology*, *28*(3), 401-414. https://doi.org/10.1016/j.euroneuro.2017.12.015.

18. *Chatzittofis, A., Nordström, P., Hellström, C., Arver, S., Åsberg, M., & Jokinen, J. (2013). CSF 5-HIAA, cortisol and DHEAS levels in suicide attempters. *European Neuropsychopharmacology,* 23, 1280–1287. . https://doi.org/10.1016/j.euroneuro.2013.02.002.

19. *Chong, S. A., Lee, W. L., Tan, C. H., Tay, A. H., Chan, A. O., & Tan, E. C. (2000). Attempted suicide and polymorphism of the serotonin transporter gene in Chinese patients with schizophrenia. *Psychiatry Research*, 97 (2–3), 101–106.

20. *Clark, D. B. (2003). Serum tryptophan ratio and suicidal behavior in adolescents: a prospective study. *Psychiatry Research*, *119*(3), 199. https://doi.org/10.1016/S0165-1781(03)00104-5

21. *Coryell, W. (1990). DST abnormality as a predictor of course in major depression. *Journal of Affective Disorders*, 19, 163–169.

22. *Coryell, W., & Schlesser, M. (2001). The dexamethasone suppression test and suicide prediction.

23. *Coryell, W., & Schlesser, M. A. (1981). Suicide and the dexamethasone suppression test in unipolar depression. *The American Journal of Psychiatry*, 138, 1120–1121.

24. * Coryell, W., & Schlesser, M. (2007). Combined biological tests for suicide prediction. *Psychiatry Research*, *150*(2), 187–191. https://doi.org/10.1016/j.psychres.2006.01.021

25. *Courtet P, Picot M-C, Bellivier F, et al. Serotonin transporter gene may be involved in short-term risk of subsequent suicide attempts. *Biological Psychiatry*. 2004;55(1):46. doi:10.1016/j.biopsych.2003.07.004.

26. * Courtet, P., Baud, P., Abbar, M., Boulenger, J. P., Castelnau, D., Mouthon, D., … Buresi, C. (2001). Association between violent suicidal behavior and the low activity allele of the serotonin transporter gene. *Molecular Psychiatry*, *6*(3), 338. https://doi.org/10.1038/sj.mp.4000856

27. *Crawford, J., Sutherland, G. R., & Goldney, R. D. (2000). No evidence for association of 5- HT2A receptor polymorphism with suicide. *American Journal of Medical Genetics*, 96, 879–880.

28. * Czyz, E. K., Berona, J. and King, C. A. (2015), A Prospective Examination of the Interpersonal‐Psychological Theory of Suicidal Behavior Among Psychiatric Adolescent Inpatients. Suicide Life Threat Behav, 45: 243-259. doi:10.1111/sltb.12125

29. *Dahlsgaard, K. K., Beck, A. T., & Brown, G. K. (1998). Inadequate response to therapy as a predictor of suicide. *Suicide and Life- Threatening Behavior*, 28, 197–204.

30. *Dieserud, Espen Røysamb, Marc T. Braverman, Odd Steffen Dalgard & Øivind Ekeberg (2003) Predicting Repetition of Suicide Attempt: A Prospective Study of 50Suicide Attempters, Archives of Suicide Research, 7:1, 1-15,DOI: 10.1080/13811110301571

31. *Du, L., Faludi, G., Palkovits, M., Demeter, E., Bakish, D., Lapierre, Y. D., . . . Hrdina, P. D. (1999). Frequency of long allele in serotonin transporter gene is increased in depressed suicide victims. *Biological Psychiatry*, 46, 196–201.

32. * Du, L. , Bakish, D. , Lapierre, Y. D., Ravindran, A. V. and Hrdina, P. D. (2000), Association of polymorphism of serotonin 2A receptor gene with suicidal ideation in major depressive disorder. Am. J. Med. Genet., 96: 56-60. doi:10.1002/(SICI)1096-8628(20000207)96:1<56::AID-AJMG12>3.0.CO;2-L

33. *Du, J., Zhang, Y., Luo, J., Jia, Y., Wei, Q., Tao, C., & Xu, H. (2018). Extracting psychiatric stressors for suicide from social media using deep learning. *BMC medical informatics and decision making*, *18*(2), 43.

34. *Ellison, L. F., & Morrison, H. I. (2001). Low serum cholesterol concentration and risk of suicide. *Epidemiology* (Cambridge, Mass.), 12,168–172.

35. *Engström, G., Alling, C., Blennow, K., Regnéll, G., & Träskman-Bendz, L. (1999). sReduced cerebrospinal HVA concentrations and HVA/5-HIAA ratios in suicide attempters. Monoamine metabolites in 120 suicide attempters and 47 controls. *European* Neuropsychopharmacology, 9, 399–405.

36. *Fiedorowicz, J. G., & Coryell, W. H. (2007). Cholesterol and suicide attempts: A prospective study of depressed inpatients. *Psychiatry Research*, 152, 11–20. https://doi.org/10.1016/j.psychres.2006.09.003

37. *Giletta, M., Hastings, P., Rudolph, K., Bauer, D., Nock, M., & Prinstein, M. (2017). Suicide ideation among high-risk adolescent females: Examining the interplay between parasympathetic regulation and friendship support. *Development and Psychopathology,* *29*(4), 1161-1175. doi:10.1017/S0954579416001218.

38. *Gorwood, P., Batel, P., Adès, J., Hamon, M., & Boni, C. (2000). Serotonin transporter gene polymorphisms, alcoholism, and suicidal behavior. Biological Psychiatry, 48, 259–264. https://doi.org/10.1016/S0006-3223(00)00840-4.

39.*Handley, T. E., Kay-Lambkin, F. J., Baker, A. L., Lewin, T. J., Kelly, B. J., Inder, K. J., ... & Kavanagh, D. J. (2016). Investigation of a suicide ideation risk profile in people with co-occurring depression and substance use disorder. *The Journal of nervous and mental disease*, *204*(11), 820-826. https://doi.org/0.1097/NMD.0000000000000473.

40. *Fawcett, J., Scheftner, W. A., Fogg, L., Clark, D. C., Young, M. A., Hedeker, D., & Gibbons, R. (1990). Time-related predictors of suicide in major affective disorder. *The American Journal of Psychiatry,* 147, 1189–1194.

41. * Fulginiti, A., He, A. S., & Negriff, S. (2018). Suicidal because I don’t feel connected or vice versa? A longitudinal study of suicidal ideation and connectedness among child welfare youth. *Child abuse & neglect*, *86*, 278-289. https://doi.org/10.1016/j.chiabu.2018.10.010

42. *Fernandes, A. C., Dutta, R., Velupillai, S., Sanyal, J., Stewart, R., & Chandran, D. (2018). Identifying suicide ideation and suicidal attempts in a psychiatric clinical research database using natural language processing. *Scientific reports*, *8*(1), 7426. https://doi.org/10.1038/s41598-018-25773-2

43. * George, S. E., Page, A. C., Hooke, G. R., & Stritzke, W. G. K. (2016). Multifacet assessment of capability for suicide: Development and prospective validation of the Acquired Capability With Rehearsal for Suicide Scale. *Psychological Assessment, 28*(11), 1452-1464. http://dx.doi.org/10.1037/pas0000276

44. * Gunn III, J. F., Goldstein, S. E., & Gager, C. T. (2018). A longitudinal examination of social connectedness and suicidal thoughts and behaviors among adolescents. *Child and Adolescent Mental Health*, *23*(4), 341-350. https://doi.org/10.1111/camh.12281

45. * Hill, R. M., Oosterhoff, B., & Kaplow, J. B. (2017). Prospective identification of adolescent suicide ideation using classification tree analysis: Models for community-based screening. *Journal of Consulting and Clinical Psychology, 85*(7), 702-711.

46. *Heffer, T., & Willoughby, T. (2017). A count of coping strategies: A longitudinal study investigating an alternative method to understanding coping and adjustment. *PloS one*, *12*(10), e0186057. https://doi.org/10.1371/journal.pone.0186057.

47. *Holma, K. M., Haukka, J., Suominen, K., Valtonen, H. M., Mantere, O., Melartin, T. K. ,... Isometsä, E. T. (2014). Differences in incidence of suicide attempts between bipolar I and II disorders and major depressive disorder. *Bipolar Disorders*, 16, 652–661. http://dx.doi.org/10.1111/bdi.12195.

48. *Holma, K. M., Melartin, T. K., Haukka, J., Holma, I. A. K., Sokero, T. P., & Isometsä, E. T. (2010). Incidence and predictors of suicide attempts in DSM–IV major depressive disorder: A five-year prospective study. *The American Journal of Psychiatry*, 167, 801–808. http://dx.doi.org/10.1176/appi.ajp.2010.09050627.

49. *Homan, C., Johar, R., Liu, T., Lytle, M., Silenzio, V., & Alm, C. O. (2014, June). Toward macro-insights for suicide prevention: Analyzing fine-grained distress at scale. In *Proceedings of the Workshop on Computational Linguistics and Clinical Psychology: From Linguistic Signal to Clinical Reality* (pp. 107-117).

50. *Huth-Bocks, A. C., Kerr, D. C. R., Ivey, A. Z., Kramer, A. C., & King, C. A. (2007). Assessment of psychiatrically hospitalized suicidal adolescents: Self-report instruments as predictors of suicidal thoughts and behavior. *Journal of the American Academy of Child & Adolescent Psychiatry*, 46, 387–395. http://dx.doi.org/10.1097/chi.0b013e31802b9535

51. *Ialongo, N. S., Koenig-McNaught, A. L., Wagner, B. M., Pearson, J. L., McCreary, B. K., Poduska, J., & Kellam, S. (2004). African American children’s reports of depressed mood, hopelessness, and suicidal ideation and later suicide attempts. *Suicide and Life-Threatening Behavior,* 34, 395–407. http://dx.doi.org/10.1521/suli.34.4.395.53743.

52. *Iribarren, C., Reed, D. M., Wergowske, G., Burchfiel, C. M., & Dwyer, J. H. (1995). Serum cholesterol level and mortality due to suicide and trauma in the Honolulu Heart Program. *Archives of Internal Medicine*, 155, 695–700.

53. *Jokinen, J., & Nordström, P. (2008). HPA axis hyperactivity as suicide predictor in elderly mood disorder inpatients. *Psychoneuroendocrinology*, 33, 1387–1393. http://dx.doi.org/10.1016/j.psyneuen.2008.07.012

54. *Jokinen, J., Carlborg, A., Mårtensson, B., Forslund, K., Nordström, A.-L., & Nordström, P. (2007). DST non-suppression predicts suicide after attempted suicide. *Psychiatry Research*, 150, 297–303. http://dx.doi.org/10.1016/j.psychres.2006.12.001

55. *Jokinen, J., Nordström, A.-L., & Nordström, P. (2009). Cerebrospinal fluid monoamine metabolites and suicide. *Nordic Journal of Psychiatry*, 63, 276–279. http://dx.doi.org/19034712

56. *Jokinen, J., Nordström, A.-L., & Nordström, P. (2009). CSF 5-HIAA and DST non-suppression—Orthogonal biologic risk factors for suicide in male mood disorder inpatients. *Psychiatry Research*, 165, 96–102. http://dx.doi.org/10.1016/j.psychres.2007.10.007

57. *Jung, J. S., Park, S. J., Kim, E. Y., Na, K. S., Kim, Y. J., & Kim, K. G. (2019). Prediction models for high risk of suicide in Korean adolescents using machine learning techniques. *PLoS one*, *14*(6).

58. *Just, M. A., Pan, L., Cherkassky, V. L., McMakin, D. L., Cha, C., Nock, M. K., & Brent, D. (2017). Machine learning of neural representations of suicide and emotion concepts identifies suicidal youth. *Nature human behaviour*, *1*(12), 911-919.

59. *Keilp, J. G., Oquendo, M. A., Stanley, B. H., Burke, A. K., Cooper, T. B., Malone, K. M., & Mann, J. J. (2010). Future suicide attempt and responses to serotonergic challenge. *Neuropsychopharmacology*, 35, 1063–1072. http://dx.doi.org/10.1038/npp.2008.28

60. *Keller, F., & Wolfersdorf, M. (1993). Hopelessness and the tendency to commit suicide in the course of depressive disorders. *Crisis: The Journal of Crisis Intervention and Suicide Prevention*, 14, 173–177.

61. *Kessler, R. C., Chalker, S. A., Luedtke, A. R., Sadikova, E., & Jobes, D. A. (2020). A preliminary precision treatment rule for remission of suicide ideation. *Suicide and Life‐ Threatening Behavior*, *50*(2), 558-572.

62. *Kleiman, E. M., & Beaver, J. K. (2013). A meaningful life is worth living: Meaning in life as a suicide resiliency factor. *Psychiatry research*, *210*(3), 934-939. http://dx.doi.org/10.1016/j.psychres.2013.08.002

63. *Kleiman, E. M., & Liu, R. T. (2014a). Prospective prediction of suicide in a nationally representative sample: Religious service attendance as a protective factor. *The British Journal of Psychiatry*, 204, 262–266. http://dx.doi.org/10.1192/bjp.bp.113.128900

64. *Kleiman, E. M., Liu, R. T., & Riskind, J. H. (2014b).Integrating the interpersonal psychological theory of suicide into the depression/suicidal ideation relationship: A short-term prospective study. *Behavior Therapy*, 45, 212–221. http://dx.doi.org/10.1016/j.beth.2013.10.007

65. *Komaki, S., Nagayama, H., Ohgami, H., Takaki, H., Mori, H., & Akiyoshi, J. (2008). Prospective study of major depressive disorder with white matter hyperintensity: Comparison of patients with and without lacunar infarction. *European Archives of Psychiatry and Clinical Neuroscience,* 258, 160–164. http://dx.doi.org/10.1007/s00406-007-0769-4

66. *Kuo, W. H., Gallo, J. J., & Eaton, W. W. (2004). Hopelessness, depression, substance disorder, and suicidality—A 13-year community-based study. *Social Psychiatry and Psychiatric Epidemiology*, 39, 497–501. http://dx.doi.org/10.1007/s00127-004-0775-z

67. *Kuramoto-Crawford, S. J., Ali, M. M., & Wilcox, H. C. (2016).Parent–Child connectedness and long-term risk for suicidal ideation in a nationally representative sample of US adolescents. *Crisis*. http://dx.doi.org/10.1027/0227-5910/a000439

68. *Lamis, D. A., & Lester, D. (2012). Risk factors for suicidal ideation among African American and European American college women. Psychology of Women Quarterly, 36(3), 337-349. http://dx.doi.org/10.1177/0361684312439186

69. *Larzelere, R. E., Smith, G. L., Batenhorst, L. M., & Kelly, D. B. (1996). Predictive validity of the Suicide Probability Scale among adolescents in group home treatment. *Journal of the American Academy of Child & Adolescent Psychiatry*, 35, 166–172. http://dx.doi.org/10.1097/00004583-199602000-00009

70. *LeCloux, M., Maramaldi, P., Thomas, K. A., & Wharff, E. A. (2017). A longitudinal study of health care resources, family support, and mental health outcomes among suicidal adolescents. *Analyses of Social Issues and Public Policy*, *17*(1), 319-338. https://doi.org/10.1111/asap.12139

71. *LeCloux, M., Maramaldi, P., Thomas, K., & Wharff, E. (2016). Family support and mental health service use among suicidal adolescents. *Journal of Child and Family Studies*, *25*(8), 2597-2606. https://doi.org/10.1007/s10826-016-0417-6

72. *Lewinsohn, P. M., Rohde, P., & Seeley, J. R. (1994). Psychosocial risk factors for future adolescent suicide attempts. *Journal of Consulting and Clinical Psychology*, 62, 297–305.

73. *Lewinsohn, P. M., Rohde, P., Seeley, J. R., & Baldwin, C. L. (2001). Gender differences in suicide attempts from adolescence to young adulthood. *Journal of the American Academy of Child & Adolescent Psychiatry*, 40, 427–434. https://doi.org/10.1097/00004583-200104000-00011

74. *Lewis, M. D., Hibbeln, J. R., Johnson, J. E., Lin, Y. H., Hyun, D. Y., & Loewke, J. D. (2011). Suicide deaths of active-duty US military and omega-3 fatty-acid status: A case-control comparison. *The Journal of Clinical Psychiatry*, 72, 1585–1590. https://doi.org/

75. * Mars, B., Heron, J., Klonsky, E. D., Moran, P., O'Connor, R. C., Tilling, K., ... & Gunnell, D. (2019). What distinguishes adolescents with suicidal thoughts from those who have attempted suicide? A population‐based birth cohort study. *Journal of child psychology and psychiatry*, *60*(1), 91-99. https://doi.org/10.1111/jcpp.12878

76. *May, A. M., Klonsky, E. D., & Klein, D. N. (2012). Predicting future suicide attempts among depressed suicide ideators: A 10-year longitudinal study. *Journal of Psychiatric Research*, 46, 946–952. https://doi.org/10.4088/JCP.11m06879

77. *Metzger, M., Tvardik, N., Gicquel, Q., Bouvry, C., Poulet, E., & Potinet‐Pagliaroli, V. (2017). Use of emergency department electronic medical records for automated epidemiological surveillance of suicide attempts: a French pilot study. *International journal of methods in psychiatric research*, *26*(2), e1522. https://doi.org/ 10.1002/mpr.1522

78. *Miller, A. B., Esposito‐Smythers, C., & Leichtweis, R. N. (2016). A short‐term, prospective test of the interpersonal–psychological theory of suicidal ideation in an adolescent clinical sample. *Suicide and Life‐Threatening Behavior*, *46*(3), 337-351. https://doi.org/10.1111/sltb.12196

79. *Miranda, R., Gallagher, M., Bauchner, B., Vaysman, R., & Marroquín, B. (2012). Cognitive inflexibility as a prospective predictor of suicidal ideation among young adults with a suicide attempt history. *Depression and Anxiety*, 29, 180–186. https://doi.org/10.1002/da.20915

80. *Morrison, R., & O’Connor, R. C. (2008). The role of rumination, attentional biases and stress in psychological distress. *British Journal of Psychology*, 99, 191–209. https://doi.org/ 10.1348/000712607X216080

81. *Mustanski, B., & Liu, R. (2013). A longitudinal study of predictors of suicide attempts among lesbian, gay, bisexual, and transgender youth. *Archives of Sexual Behavior*, 42, 437–448. https://doi.org/10.1007/s10508-012-0013-9

82. *Nielsen, M. B., Nielsen, G. H., Notelaers, G., & Einarsen, S. (2015). Workplace bullying and suicidal ideation: a 3-wave longitudinal Norwegian study. *American Journal of Public Health*, *105*(11), e23-e28. https://doi.org/10.2105/AJPH.2015.302855

83. *Niméus, A., Alsén, M., & Träskman-Bendz, L. (2000). The suicide assessment scale: An instrument assessing suicide risk of suicide attempters. *European Psychiatry*, 15, 416–423.

84. *Niméus, A., Träskman-Bendz, L., & Alsén, M. (1997). Hopelessness and suicidal behavior. *Journal of Affective Disorders*, 42, 137–144.

85. *Nordentoft, M., Breum, L., Munck, L. K., Nordestgaard, A. G., Hunding, A., & Laursen Bjaeldager, P. A. (1993). High mortality by natural and unnatural causes: A 10 year follow up study of patients admitted to a poisoning treatment centre after suicide attempts. *BMJ* (Clinical Research Ed.), 306,1637–1641.

86. *Nordström, P., Samuelsson, M., Asberg, M., Träskman-Bendz, L., Aberg-Wistedt, A., Nordin, C., & Bertilsson, L. (1994). CSF 5-HIAA predicts suicide risk after attempted suicide. *Suicide and Life-Threatening Behavior*, 24, 1–9.

87. *O'Connor, R. C., Smyth, R., Ferguson, E., Ryan, C., & Williams, J. M. (2013). Psychological processes and repeat suicidal behavior: A four-year prospective study. *Journal of consulting and clinical psychology*, *81*(6), 1137. https://doi.org/10.1037/a0033751

88. *Oh, B., Yun, J. Y., Yeo, E. C., Kim, D. H., Kim, J., & Cho, B. J. (2020). Prediction of suicidal ideation among Korean adults using machine learning: a cross-sectional study. *Psychiatry investigation*, *17*(4), 331.

89. *Pallaskorpi, S., Suominen, K., Ketokivi, M., Valtonen, H., Arvilommi, P., Mantere, O., ... & Isometsä, E. (2017). Incidence and predictors of suicide attempts in bipolar I and II disorders: A 5‐year follow‐up study. *Bipolar disorders*, *19*(1), 13-22. https://doi.org/10.1111/bdi.12464

90. *Panagioti, M., Gooding, P. A., & Tarrier, N. (2015). A prospective study of suicidal ideation in posttraumatic stress disorder: the role of perceptions of defeat and entrapment. *Journal of clinical psychology*, *71*(1), 50-61. https://doi.org/10.1002/jclp.22103

91. *Passos, I. C., Mwangi, B., Cao, B., Hamilton, J. E., Wu, M. J., Zhang, X. Y., ... & Soares, J. C. (2016). Identifying a clinical signature of suicidality among patients with mood disorders: A pilot study using a machine learning approach. *Journal of affective disorders*, *193*, 109-116.

92. *Podlogar, T., Žiberna, J., Poštuvan, V., & CR Kerr, D. (2017). Belongingness and burdensomeness in adolescents: Slovene translation and validation of the Interpersonal Needs Questionnaire. *Suicide and Life‐Threatening Behavior*, *47*(3), 336-352. https://doi.org/10.1111/sltb.12276

93. *Puzia, M. E., Kraines, M. A., Liu, R. T., & Kleiman, E. M. (2014). Early life stressors and suicidal ideation: mediation by interpersonal risk factors. *Personality and individual differences*, *56*, 68-72. https://doi.org/10.1016/j.paid.2013.08.027

94. *Qiu, T., Klonsky, E. D., & Klein, D. N. (2017). Hopelessness predicts suicide ideation but not attempts: A 10‐year longitudinal study. *Suicide and Life‐Threatening Behavior*, *47*(6), 718-722. https://doi.org/ 10.1111/sltb.12328

95. *Quiñones, V., Jurska, J., Fener, E., & Miranda, R. (2015). Active and passive problem solving: Moderating role in the relation between depressive symptoms and future suicidal ideation varies by suicide attempt history. *Journal of clinical psychology*, *71*(4), 402-412. https://doi.org/10.1002/jclp.22155

96. *Ribeiro, J. D., Pease, J. L., Gutierrez, P. M., Silva, C., Bernert, R. A., Rudd, M. D., & Joiner Jr, T. E. (2012). Sleep problems outperform depression and hopelessness as cross- sectional and longitudinal predictors of suicidal ideation and behavior in young adults in the military. *Journal of affective disorders*, *136*(3), 743-750. https://doi.org/10.1016/j.jad.2011.09.049

97. *Ribeiro, J. D., Yen, S., Joiner, T., & Siegler, I. C. (2015). Capability for suicide interacts with states of heightened arousal to predict death by suicide beyond the effects of depression and hopelessness. *Journal of affective disorders*, *188*, 53-59. https://doi.org/10.1016/j.jad.2015.07.037

98. *Riihimäki, K., Vuorilehto, M., Melartin, T., Haukka, J., & Isometsä, E. (2014). Incidence and predictors of suicide attempts among primary-care patients with depressive disorders: A 5-year prospective study. *Psychological Medicine*, 44, 291–302. https://doi.org/ 10.1017/S0033291713000706

99. *Robinson, J., Harris, M. G., Harrigan, S. M., Henry, L. P., Farrelly, S., Prosser, A. ,... McGorry, P. D. (2010). Suicide attempt in first-episode psychosis: A 7.4 year follow-up study. *Schizophrenia Research*, 116, 1-8. https://doi.org/ 10.1016/j.schres.2009.10.009

100. * Roeder, K. M., & Cole, D. A. (2018). Simultaneous Longitudinal Examination of Hopelessness, Thwarted Belongingness, and Perceived Burdensomeness as Predictors of Suicide Ideation. *Suicide and Life‐Threatening Behavior*. https://doi.org/10.1111/sltb.12508

101. *Roy, A. (1992). Hypothalamic-pituitary-adrenal axis function and suicidal behavior in depression. *Biological Psychiatry*, 32, 812–816.

102. *Roy, A., Agren, H., Pickar, D., Linnoila, M., Doran, A. R., Cutler, N. R., & Paul, S. M. (1986). Reduced CSF concentrations of homovanillic acid and homovanillic acid to 5-hydroxyindoleacetic acid ratios in depressed patients: Relationship to suicidal behavior and dexamethasone nonsuppression. The American Journal of Psychiatry, 143, 1539–1545.

103. *Ryu, S., Lee, H., Lee, D. K., & Park, K. (2018). Use of a machine learning algorithm to predict individuals with suicide ideation in the general population. *Psychiatry investigation*, *15*(11), 1030. https://doi.org/ 10.30773/pi.2018.08.27

104. *Samuelsson, M., Jokinen, J., Nordström, A. L., & Nordström, P. (2006). CSF 5-HIAA, suicide intent and hopelessness in the prediction of early suicide in male high-risk suicide attempters. *ActaPsychiatrica Scandinavica*, 113, 44–47. https://doi.org/10.1111/j.1600-0447.2005.00639.x

105. *Sanderson, M., Bulloch, A. G., Wang, J., Williamson, T., & Patten, S. B. (2019). Predicting death by suicide using administrative health care system data: Can feedforward neural network models improve upon logistic regression models?. *Journal of affective disorders*, *257*, 741-747.

106. *Sher, L., Carballo, J., Grunebaum, M. F., Burke, A. K., Zalsman, G., Huang, Y. Y.,...Oquendo, M. A. (2006). A prospective study of the association of cerebrospinal fluid monoamine metabolite levels with lethality of suicide attempts in patients with bipolar disorder. *Bipolar Disorders*, 8, 543–550. https://doi.org/ 10.1111/j.1399-5618.2006.00319.x

107. * Shi, P., Ren, H., Li, H., & Dai, Q. (2018). Maternal depression and suicide at immediate prenatal and early postpartum periods and psychosocial risk factors. *Psychiatry research*, *261*, 298-306. https://doi.org/10.1016/j.psychres.2017.12.085

108. *Sigurdson, J. F., Undheim, A. M., Wallander, J. L., Lydersen, S., & Sund, A. M. (2018). The Longitudinal Association of Being Bullied and Gender with Suicide Ideations, Self‐Harm, and Suicide Attempts from Adolescence to Young Adulthood: A Cohort Study. *Suicide and Life‐Threatening Behavior*, *48*(2), 169-182. https://doi.org/ 10.1111/sltb.12358

109. *Sokero, T. P., Melartin, T. K., Rytsälä, H. J., Leskelä, U. S., Lestelä-Mielonen, P. S., & Isometsä, E. T. (2005). Prospective study of risk factors for attempted suicide among patients with DSM–IV major depressive disorder. The British Journal of Psychiatry, 186, 314–318. https://doi.org/10.1192/bjp.186.4.314

110. *Stange, J. P., Hamilton, J. L., Burke, T. A., Kleiman, E. M., O’Garro-Moore, J. K., Seligman, N.D., ... & Alloy, L. B. (2015). Negative cognitive styles synergistically predict suicidal ideation in bipolar spectrum disorders: A 3-year prospective study. *Psychiatry research*, *226*(1), 162-168. https://doi.org/10.1016/j.psychres.2014.12.042

111. *Targum, S. D., Rosen, L., & Capodanno, A. E. (1983). The dexamethasone suppression test in suicidal patients with unipolar depression. *The American Journal of Psychiatry*, 140, 877–879.

112. *Träskman, L., Åsberg, M., Bertilsson, L., & Sjöstrand, L. (1981). Monoamine metabolites in CSF and suicidal behavior. *Archives of General Psychiatry*, 38, 631–636.

113. *Troister, T., Davis, M. P., Lowndes, A., & Holden, R. R. (2013). A five-month longitudinal study of psychache and suicide ideation: Replication in general and high-risk university students. *Suicide and Life-Threatening Behavior*, 43, 611–620. https://doi.org/10.1111/sltb.12043

114. *Valtonen, H. M., Suominen, K., Mantere, O., Leppämäki, S., Arvilommi, P., & Isometsä, E. T. (2006). Prospective study of risk factors for attempted suicide among patients with bipolar disorder. *Bipolar Disorders*, 8, 576–585. https://doi.org/10.1192/bjp.186.4.314

115. *Van Orden, K. A., Wiktorsson, S., Duberstein, P., Berg, A. I., Fässberg, M. M., & Waern, M. (2015). Reasons for attempted suicide in later life. *The American Journal of Geriatric Psychiatry*, *23*(5), 536-544. https://doi.org/10.1016/j.jagp.2014.07.003

116. *Walsh, C. G., Ribeiro, J. D., & Franklin, J. C. (2018). Predicting suicide attempts in adolescents with longitudinal clinical data and machine learning. *Journal of child psychology and psychiatry*, *59*(12), 1261-1270. https://doi.org/10.1111/jcpp.12916

117. * Wang, Y. , Bhaskaran, J. , Sareen, J. , Wang, J. , Spiwak, R. & Bolton, J. M. (2015). Predictors of Future Suicide Attempts Among Individuals Referred to Psychiatric Services in the Emergency Department. The Journal of Nervous and Mental Disease, 203(7), 507–513. doi: 10.1097/NMD.0000000000000320.

118. *Wedig, M., Silverman, M. H., Frankenburg, F. R., Reich, D. B., Fitzmaurice, G., & Zanarini, M. C. (2012). Predictors of suicide attempts in patients with borderline personality disorder over 16 years of prospective follow-up. *Psychological medicine*, *42*(11), 2395-2404. https://doi.org/10.1017/S0033291712000517

119. *Wilcox, H, Arria, A. M., Caldeira, K. M., Vincent, K. B., Pinchevsky, G. M., & O’Grady, K. E. (2010). Prevalence and predictors of persistent suicide ideation, plans, and attempts during college. *Journal of Affective Disorders*, 127, 287–294. https://doi.org/10.1016/j.jad.2010.04.017

120. *Wilkinson, P., Kelvin, R., Roberts, C., Dubicka, B., & Goodyer, I. (2011). Clinical and psychosocial predictors of suicide attempts and nonsuicidal self-injury in the Adolescent Depression Antidepressants and Psychotherapy Trial (ADAPT). *American journal of psychiatry*, *168*(5), 495-501. https://doi.org/ https://doi.org/10.1176/appi.ajp.2010.10050718

121. *Yerevanian, B. I., Feusner, J. D., Koek, R. J., & Mintz, J. (2004). The dexamethasone suppression test as a predictor of suicidal behavior in unipolar depression. *Journal of Affective Disorders*, 83, 103–108. https://doi.org/10.1016/j.jad.2004.08.009

122. *Yerevanian, B. I., Olafsdottir, H., Milanese, E., Russotto, J., Mallon, P., Baciewicz, G., & Sagi, E. (1983). Normalization of the dexamethasone suppression test at discharge from hospital: Its prognostic value. *Journal of Affective Disorders*, 5, 191–197. https://doi.org/10.1016/0165-0327(83)90041-1

123. *Young, M. A., Fogg, L. F., Scheftner, W., Fawcett, J., Akiskal, H., & Maser, J. (1996). Stable trait components of hopelessness: Baseline and sensitivity to depression. *Journal of Abnormal Psychology*, 105,155–165.

124. *Zhang, Y., Wu, C., Yuan, S., Xiang, J., Hao, W., & Yu, Y. (2018). Association of aggression and suicide behaviors: A school-based sample of rural Chinese adolescents. *Journal of affective disorders*, *239*, 295-302. https://doi.org/10.1016/j.jad.2018.07.029

125. *Zureik, M., Courbon, D., & Ducimetière, P. (1996). Serum cholesterolconcentration and death from suicide in men: Paris prospective study I. *BMJ* (Clinical Research Ed.), 313, 649–651.
