# Supplementary material for: A direct comparison of theory-driven and machine learning prediction of suicide: A meta-analysis
Source: PLoS One. 2021 Apr 12;16(4):e0249833. doi: 10.1371/journal.pone.0249833 (PMC8041204; doi:10.1371/journal.pone.0249833)
Supplement: Attachment — Submitted filename: 20.11.20 PONE-D-20-21525_review - Yaakov Ophir.pdf [file pone.0249833.s006.pdf]

Review on Manuscript PONE-D-20-21525

A Head-to-Head Comparison of Theory Driven vs Machine Learning Prediction of Suicide: A Meta-Analysis

Yaakov Ophir

Thank you for providing me the opportunity to review this valuable research. To my understanding, the findings of this study are extremely important to the future development of suicide prediction models that could help reduce this tragic phenomenon. However, precisely because I believe that the study is important, I encourage the authors to conduct major revisions in the current version of the manuscript (see below). Please know that I do not sanctify my own recommendations for modifications and that it is my unequivocal recommendation to the editor to accept this manuscript. However, I do have multiple queries/suggestions, that, in my opinion, can significantly contribute to the clarity of the narrative of the article and the transmission of its main goals to the scientific community.

Abstract

1. To date, a direct, head-to-head comparison…

On the one hand, this is a very direct (and therefore informative) description of the research goal. On the other hand, it sounds a bit argumentative/competitive, and I am not sure that this is the most appropriate/tactically-smart narrative. You can consider re-framing it to something like: ML approaches emerged and are said to yield improved predictions but to date, this argument has not been investigated empirically in a systematic manner. The present... a meta-analysis, which allows a direct comparison (without the "head-to-head" phrase).

2. Conclusions: Among traditional theories, Ideation-to-Action outperformed Biological, Hopelessness, and BioSocial theories of suicide

It is strange to mention a conclusion that is not preceded by relevant findings in the Results section of the abstract. Also, are you sure that Ideation-to-Action outperformed Hopelessness? see a more detailed comment in the Results section.

Introduction

3. kills nearly one million people every year

According to the WHO, "Close to 800 000 people die due to suicide every year". See in: https://www.who.int/news-room/fact-sheets/detail/suicide#:~:text=Close%20to%20800%20000%20people,%2D19%2Dyear%2Dolds.

4. suicide rates have failed to appreciably decline in the United States

I think you should decide whether you want to discuss suicide as a "global epidemic" or as a US phenomenon. If the global framing is used, then, this sentence should be re-phrased or deleted, because the overall picture is mixed. In fact, suicide rates in many many countries have actually fallen! See in:

https://ourworldindata.org/suicide#how-have-suicide-rates-changed

5. Investigations into suicide have traditionally… based on one biopsychosocial factor or a small set of factors combined together.

Is this the main (and only) difference between the traditional methods and ML methods? If so, it sounds a bit too easy, almost making the research question (what is the superior method) redundant. Of course, any analysis that comprises several predictors will be better than an analysis that has only one predictor. If, indeed, this is the only difference, perhaps you can consider adding a sentence regarding the unique value of using top-down, theory driven predictors in the traditional methods, so that the research problem will be a little bit more complex/interesting.

6. The two competing approaches have yet to be directly compared, head-to-head…

See comment 1, above. I think the point is not the lack of head-to-head comparison but a lack of a systematic empirical evidence that ML is indeed a desirable method in this line of research.

7. …Theories of Suicide all cluster into (3) the Ideation-to-Action perspective of suicide.

If these theories focus on the shift from ideation to action, then they may not be suitable to the prediction of suicide ideation (only to attempts). See also comment 21.

8. However, some recent meta-analyses of risk factors—namely…—have determined that biopsychosocial constructs are suboptimal predictors of STB outcomes.

This is a very important statement. However, it does not speak directly with the previous sentence. The four theories have achieved empirical support, however (all kinds of) risk factors are suboptimal. But are these risk factors part of the four theories? The reader does not know. Also, the following sentence says that the four theoretical approaches were not investigated. Thus, leaving the reader a bit perplexed.

9. Franklin et al., 2016

Should be 2017.

10. The first aim of the present study…

The previous sentence regarding the suboptimal predictors (Franklin et al., 2017) is not really required to present the first aim of the study (comparing the efficacy of the four theoretical approaches). It is more relevant to the second (and from what I understand, the main) goal of the study (proving the importance of ML methods).

This also makes the narrative a bit awkward. You may consider breaking the rational into two separate gaps/goals (first, several theories were proposed but with no direct comparison, therefore goal 1. Second, suboptimal predictors can be overcome by using ML - but this is also not proven systematically, therefore goal 2). Alternatively, you can present the two gaps and then present two consecutive goals.

11. Models generally use large datasets…

'Models' can refer to traditional statistical analysis as well (e.g., regression model). Please consider adding the term machine learning before the word 'model'. You can also consider using the conventional acronym (ML) throughout the paper.

12. (see Belsher et al., 2019)

Two following highly relevant meta-analyses are missing in the current version of the paper:

Burke, T. A., Ammerman, B. A., & Jacobucci, R. (2019). The use of machine learning in the study of suicidal and non-suicidal self-injurious thoughts and behaviors: A systematic review. Journal of affective disorders, 245, 869-884.

Bernert, R. A., Hilberg, A. M., Melia, R., Kim, J. P., Shah, N. H., & Abnousi, F. (2020). Artificial intelligence and suicide prevention: a systematic review of machine learning investigations. International journal of environmental research and public health, 17(16), 5929.

13. Thus it is unclear if the accurate novel prediction of machine learning is any improvement upon traditional prediction.

In my opinion, this is a more elegant way to present the gap than the 'head-to-head' phrasing that was used in the abstract.

14. The present work represents… of these two competing approaches

These are **not** really competing approaches. Indeed, the current work place the two approaches, one against the other ('head to head), but this does not mean that the approaches are competing, let alone, contradicting.

15. One possibility is that traditional approaches… improve only slightly… An alternative possibility is that theoretical constructs demonstrate strong prediction… consistent with existing theories.

Not sure what is the role of these two sentences. First, there is a third option (that traditional approach will not improve the prediction beyond general risk factors). Second, these sentences are not posited as a directional hypothesis (which one of the two options is more reasonable). Third, these options are not backed up with references. So that, in practice, these sentences are not adding any new information beyond the open question (whether the theoretical approaches are valuable or not for suicide prediction).

16. The present work also aims to identify characteristics of highly accurate machine learning models.

This is an important goal. However, it comes as a surprise to the reader, as a pleasant 'side effect' of the research. If indeed, you are planning to achieve this goal, please consider building a designated

rational and explicitly declare that you are planning to achieve **three** goals in this study (also in the abstract - currently this goal has no trace in the abstract).

Method

17. Literature searches… were conducted until prior to May 1, 2020

I have received the manuscript on November 14, 2020. This is quite a long gap. Of course, I do not expect that a new search and a new analysis will be conducted in order to complement these missing, more than six months. However, if you do consider doing that, it would significantly contribute to the comprehensiveness of the study, especially in light of the fact that this field (ML in suicide research) is growing very fast.

18. Finally, we searched the grey literature…

Should this expression be explained for readers who are less familiar with meta-analysis conventional practices? (a short description in parenthesis will do the work)

19. Given the paucity of literature…

This is (as will be mentioned in the discussion section) a noteworthy weakness of the study. Perhaps you can elaborate a little about the inherent paradigm of ML methods that distinguish between learning data and test data and on the fact that predictions are made on 'new' unseen data.
Indeed, this does not replace the need for longitudinal ML studies, but it distinguishes ML from traditional studies. I believe that it can be asserted that the 'power' of the results from cross-sectional ML designs is different than (superior to) the power of the results from cross-sectional traditional designs. This is because the meaning of the term 'prediction' is somewhat different between the two approaches.

Results

20. Ideation-to-Action. For overall prediction (**wOR = 2.41**…)

The overall prediction of ideation to action (wOR) is said to be 2.41 but the specific predictions were all significantly lower (ideation = 2.12, attempts = 1.52, and deaths = 0.96). Does this make sense? This is not the case in the other theoretical approaches (in which the overall figure seems more like a weighted average of the three suicide outcomes). If the overall prediction of ideation to action is not correct, this affects the conclusions of the study and should be fixed throughout the manuscript.

21. Ideation-to-Action predicted ideation (wOR = 2.12…)

As mentioned above in comment 7, it is unclear how can a theory that focus on the shift from ideation to action predict ideation?

22. Analyses indicated a significant improvement in predictive ability across time **when traditional** (b = .023, p < .001) **and but** not within machine learning studies (b =.03, p= 0.17) were analyzed independently.

Something is wrong in this sentence.

23. *Prediction across theories*.

The paragraph that follows this subtitle does not add new information beyond the results that were presented above.

24. *Prediction across suicide outcomes.*

Not sure whether the paragraph that follows this subtitle adds new information to the results presented above. I do however understand the importance of showing that the CI of the different approaches do not overlap. Perhaps the authors can consider integrating these comparisons within the paragraph that presents the results of the ML approach.

25. Findings indicated that machine learning models predicted suicidal ideation (…), suicide attempts (…), and suicide death (…).

This sentence is not completed. I think the authors meant that ML models predicted suicidal... and suicide death **better than traditional approaches**.

26. *Positive cases in model testing*.

The paragraph that follows this subtitle is hard to follow. The goal of the analysis ("investigate the behavior of machine learning models") is obscure and there is no clear rational for this analysis in the introduction. I actually think that this part may be very important but in its current status it only disturbs the reading. Please consider improving this section (and its related parts in the article) or deleting it altogether.

27. First we investigated if the number of positive cases was predictive of accuracy. Findings indicated that it was not (…).

I think this last sentence (and the previous one) can be better phrased.

28. **We calculated** percentage of STB outcomes **was calculated** as follows…

Something is wrong in this sentence.

29. Analyses indicated a statistically significant relation between the proportion of STB cases and accuracy of models (b = .009, p = .0041).

This finding should be further explained. What does it mean in laymen language?

Discussion

30. Unfortunately, those meta-analyses were too broad…, thus a rush to abandon theories of suicide was premature.

Was there really such a 'rush'? If so, a citation is needed.

31. The present study first sought to summarize and evaluate extant literature regarding predictive ability of traditional theories of suicide.

A similar narrative difficulty appeared in the introduction. This 'first aim' is valid but it is not a natural extension of the previous paragraph. In my opinion, you can open the discussion section by saying that this study aimed to achieve two goals (or three if the last goal is maintained), 1 and 2 and that the findings were... Then you can move to explain why these findings are important (return to the status quo, the rush, the gaps, etc.).

32. This increased predictive accuracy… likely driven by effect sizes related to the Ideation-to-Action framework.

Please check this. Something does not add up, not in the numbers presented in the results section and not in the short rational that was supplied for this theoretical framework (see also comments 7 and 21).

33. This stands as a **victory** for theories clustered under the framework of Ideation-to-Action

To my knowledge, this terminology is less acceptable in psychological science (see my related comments on 'competitive' and 'head-to-head').

34. That is, the Interpersonal Theory… (all theories in the Ideation-to-Action framework) propose that suicidal ideation precedes serious and/or lethal suicide attempts…

Once again, if this is indeed the focus of this approach, how does it contribute to the prediction of ideation itself?

35. Even though theories… were statistically significant…, the **low base rate** of suicide calls…

This is a good point that, in my opinion, should be further explained, preferably, in the introduction. Elaborating on this point may help readers understand why the small size effects of theory driven approaches are limiting our applicative ability to detect STBs.

36. For example, few published articles contained data **sufficient** for analyses (k = 20) and when contacted most corresponding authors did not share their data.

Do you mean **insufficient**?

37. It was expected that the percentage of cases (i.e. STB positive participants…)

As mentioned above, this is an interesting point. However, it is not developed enough. Why was this the expectation (in the introduction - perhaps provide examples from other fields, perhaps discuss the base-rate problem, etc.) and how come this expectation was not realized (in the discussion). Alternatively, perhaps omit this part (and the entire third goal of the study) altogether.

38. Further exploratory moderation analyses… were not conducted as too few articles provided sufficient data.

This sounds like an 'apology' for not fulfilling an action that was not fully introduced and rationalized to begin with (in the introduction). This is another example of the incompleteness of the 'third goal' of the study.

39. Thus, it largely remains unclear why some machine learning models predict extremely well and others do not.

This is actually a good start of developing the rationale behind the third goal of the study.

40. Thus, it was unclear if, only by including longitudinal prediction, machine learning accuracy would **decrease**.

I expect that ML models will only benefit from longitudinal designs, but I understand the proposed limitation.

41. Thus, predictive accuracy—the common metric in the present meta-analysis—lends itself to the utility of machine learning models, potentially at the cost of explainability.

This is an interesting point. Please consider elaborating on it because for readers who are not familiar with ML strategies, this distinction is not intuitive - how come prediction and explainability come, one at the expanse of the other? After all, the more the variable explains the criterion, the more it is expected to contribute to its prediction.

42. A notable exception, and perhaps a middle ground to the conflict between the two approaches,

A 'middle ground' goes back to the 'war' between the two approaches. If you think that combining theory and ML worth further investigation, perhaps phrase it as such (extracting the benefits from each approach). However, please remember, that in its current form, the 'benefits' of theory-driven approaches are not provided/convincing in the introduction section.

43. However, it is unlikely that machine learning models trained and tested for example, among adolescents, will perform with high accuracy prediction among a sample of veterans.

Is this a unique limitation of ML? Is it not a problem for traditional approaches as well?

44. This suggests that **smaller samples** tend to have much larger effect sizes **than smaller** samples.

Should the second "smaller" be "larger"? (the word "smaller" appears twice in this sentence).

45. In summary, prediction of STB outcomes…

In my opinion, you do not need to provide a summary paragraph here. You can continue (from the limitation section) straight to the future directions section, especially since you have a conclusion section by the end of the article.

46. Conclusions

The conclusions regarding the first goal of the study (i.e., the effectiveness of the four theoretical approaches) are missing from this section. The third goal (which, as mentioned above, is not fully described in the manuscript) is also missing from this conclusion.

47. The key is to integrate the insights of the traditional approaches with the empirical power of the data-driven techniques.

This is the first time this recommendation is mentioned (the one that proposed the 'middle ground' from above does not imply the benefits of such integration). As mentioned above, if you think that this is an important recommendation, please consider elaborating on it earlier in the discussion section.

Dear authors,

As mentioned in the beginning of this review, please do not be discourage from this seemingly long list of comments. Feel free to disagree with my suggestions and remember that all of these comments are aimed to improve this important piece of science!

I have great appreciation to the work that was conducted in this project,

Best regards,

Yaakov
