# Supplementary material for: A direct comparison of theory-driven and machine learning prediction of suicide: A meta-analysis
Source: PLoS One. 2021 Apr 12;16(4):e0249833. doi: 10.1371/journal.pone.0249833 (PMC8041204; doi:10.1371/journal.pone.0249833)
Supplement: Attachment — Submitted filename: renamed_e4efb.docx [file pone.0249833.s007.docx]

**Review on Manuscript PONE-D-20-21525_R1**

**A Direct Comparison of Theory-Driven and Machine Learning Prediction of Suicide: A Meta-Analysis**

Yaakov Ophir

To the authors,

I thank you again for providing me the opportunity to review your work. The revised manuscript is extremely readable, and I have really enjoyed reading it. From my perspective, the article is "ready to go". In its current form, I think that the article fills an important empirical brick, which was missing from the literature that advocates the integration of Machine Learning (ML) in suicide research.

On that note, I propose that, in the final version, you would acknowledge previous works that raised concerns regarding the superiority and reliability of ML in suicide/mental health research. In a way, your work addresses these concerns (but please remember to maintain the respected tone that is now so well-integrated in the revised manuscript).

Attached are two references for example.

Siddaway, A. P., Quinlivan, L., Kapur, N., O'Connor, R. C., & de Beurs, D. (2020). Cautions, concerns, and future directions for using machine learning in relation to mental health problems and clinical and forensic risks: A brief comment on “Model complexity improves the prediction of nonsuicidal self-injury”(Fox et al., 2019).

Jacobucci, R., Littlefield, A. K., Millner, A. J., Kleiman, E. M., & Steinley, D. (2021). Evidence of Inflated Prediction Performance: A Commentary on Machine Learning and Suicide Research. *Clinical Psychological Science*, 2167702620954216.

Altogether, I believe your work is solid and highly important and I truly hope to see it published soon.

Best regards,

Yaakov
